# A Liposomal Formulation for Improving Solubility and Oral Bioavailability of Nifedipine

**DOI:** 10.3390/molecules25020338

**Published:** 2020-01-14

**Authors:** Ye Bi, Bingcong Lv, Lianlian Li, Robert J. Lee, Jing Xie, Zhidong Qiu, Lesheng Teng

**Affiliations:** 1 Key Laboratory of Effective Components of Traditional Chinese Medicine, Changchun University of Chinese Medicine, Changchun 130117, China; biye88@outlook.com; 2 Practice Training Center, Changchun University of Chinese Medicine, Changchun 130117, China; 3 School of Life Sciences, Jilin University, Changchun 130117, China; bingconglv@163.com (B.L.); lill15@mails.jlu.edu.cn (L.L.); lee.1339@osu.edu (R.J.L.); xiejing@jlu.edu.cn (J.X.); 4 Division of Pharmaceutics, College of Pharmacy, The Ohio State University, Columbus, OH 43210, USA; 5 Department of Pharmacy, Changchun University of Chinese Medicine, Changchun 130117, China

**Keywords:** nifedipine, proliposomes, pharmacokinetics, bioavailability

## Abstract

Proliposomes were used to improve the solubility and oral bioavailability of nifedipine. Nifedipine proliposomes were prepared by methanol injection-spray drying method. The response surface method was used to optimize formulation to enhance the encapsulation efficiency (EE%) of nifedipine. The particle size of nifedipine proliposomes after rehydration was 114 nm. Surface morphology of nifedipine proliposomes was observed by a scanning electron microscope (SEM) and interaction of formulation ingredients was assessed by differential scanning calorimetry (DSC). The solubility of nifedipine is improved 24.8 times after forming proliposomes. In vitro release experiment, nifedipine proliposomes had a control release effect, especially in simulated gastric fluid. In vivo, nifedipine proliposomes significantly improved the bioavailability of nifedipine. The area under the concentration-time curve (AUC_0–∞_) of nifedipine proliposomes was about 10 times than nifedipine after oral administration. The elimination half-life (T_1/2β_) of nifedipine was increased from 1.6 h to 6.6 h. In conclusion, proliposomes was a promising system to deliver nifedipine through oral route and warranted further investigation.

## 1. Introduction

4-(2-Nitrophenyl)-2,6-dimethyl-3,5-dicarbomethoxy-1,4-dihydropyridine (Nifedipine) is a calcium channel blocker drug used for the treatment of angiocardiopathy. Although oral administration is the best convenient route and has better patient compliance, bioavailability of nifedipine has been limited by poor solubility, photo-instability, or short plasma half-life [1]. Nifedipine is a BCS class II agent with a solubility of 5–6 μg/mL in the pH range from 4 to 13 [2], resulting in low bioavailability [3]. Improving solubility and membrane permeability could lead to enhance of oral bioavailability [4]. Drug solubility could be increased by a dry or wet process, for example, indissolvable nifedipine co-grinding with a carrier such as polyvinyl pyrrolidone or microcrystalline cellulose by dry method could enhance the solubility [5,6,7]. However, this method could not be used to produce Nano-level preparation. On the other hand, the wet method usually uses high-pressure homogenization to form uniform nanoparticles in the presence of an organic solvent [8]. However, it is a challenge to remove residual organic solvent. Therefore, novel formulations were also developed to improve therapeutic effects of nifedipine, including microparticles [9], inclusion complexes [10], freeze-dried lipid nanoparticles [7], and gelatin microcapsules [11,12].

In this study, proliposomes were evaluated as a carrier for nifedipine oral administration [13]. Proliposomes have the advantage of continuously releasing drug and stabilizing its blood concentration, and they may improve bioavailability and drug stability [14]. The purpose of preparing proliposomes is improving bioavailability and elimination half-life of nifedipine, which could avoid the blood pressure fluctuations caused by repeated administration and improve therapeutic effects.

## 2. Results and Discussion

### 2.1. Preparation of Nifedipine Proliposomes

Proliposomes were prepared to improve solubility and pharmacokinetic characteristics of nifedipine. Nifedipine embedded in lipid bilayers achieved high encapsulation efficiency (EE%). Chol was added into the formulation to enhance the liposome stability through altering its phase transition behavior. Spray-drying was used to prepare proliposomes because nifedipine was not very sensitive to brief exposure to high temperature [15]. We were concerned that the high temperature in the process of spray-drying might destroy the liposomal membrane, so we investigated the addition of carriers (mannitol, sorbitol, and lactose) and selected mannitol as a carrier to protect liposomes from agglomerating in the process of spray drying [15,16].

Mannitol is a highly water-soluble material (≈180 mg/mL), it has two functions in forming proliposomes by spray drying. (1) Protecting the structural integrity of liposomes upon exposure to high temperature. Mannitol could form “interstitial bridges” between liposomes for preventing irreversible interparticle fusion. (2) In the gastrointestinal tract, the “interstitial bridges” would dissolve to release liposomes [17].

### 2.2. Optimization of Proliposomes Formulation

On the basis of single factor experiments, we designed 15 groups of three-factor, three-coded level experiments to optimize proliposomes EE% with a Box–Behnken design [18]. The experimental conditions and the corresponding results were shown in Table 1. EE% of nifedipine in different prescriptions ranged from 71.93% to 94.12%. Then, we established a mathematical model for optimizing experimental factors of affecting EE% to obtain the optimal formulation. The fitting equation between EE% and three factors was

EE% = 93.86 − 4.12375 *X1* − 0.35125 *X2* − 2.6125 *X3* + 0.2525 *X1X2* − 5.6 *X1X3* − 1.865 *X2X3* − 4.92625 *X1*^2^ − 11.52125 *X2*^2^ − 4.47375 *X3*^2^ (1)

**Figure 1:**
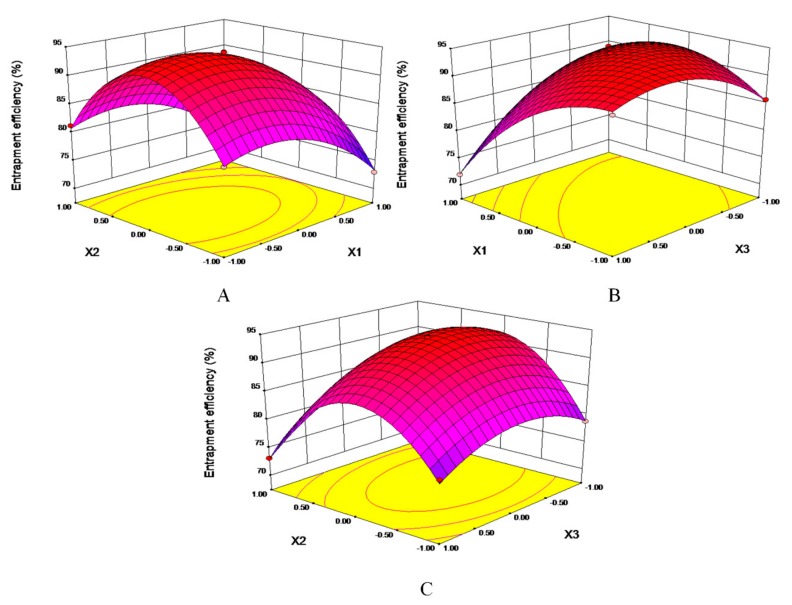
Response surface plots showing the interaction of factors. (**A**): EE% = f (*X1*, *X2*). (**B**): EE% = f (*X1*, *X3*). (**C**): EE% = f (*X2*, *X3*).

Statistical analysis of variance for the experimental results was shown in Table 2. The R^2^ of mathematical model was 99.76%, the *p*-value was less than 0.0001, and “lack of fit” was not significant (*p* = 0.2007), which meant the fit of the model was favorable and experiment error was acceptable. It indicates that the model could be used to analyze and predict EE% of nifedipine proliposomes. The linear term (*X1*, *X3*), an interaction term (X1*X3, X2*X3), and quadric entries (*X1*^2^, *X2*^2^, *X3*^2^) had significant contributions, which suggested there were not a simple linear relationship between independent variables and dependent variable but that there was a certain interaction. In Figure 1, response surface plots were used to present the interaction of factors to predict the best formulation. The three response surface plots all had convex surfaces with opening downward. Therefore, the response value of EE% has a maximum value in the range of optimization parameters.

In Figure 1B, the contour line was elliptical. There was an obvious angle between the axis of the oval and coordinate, showing that the two factors (*X1*, *X3*) had the most significant interaction. The optimal formulation was 13.92 mg/mL of SPC, 3.52 mg/mL of Chol, and 11.14 mg/mL of nifedipine dissolved in methanol. The optimal ratio of lipid phrase and water phrase was 1:8, the mass ratio of SPC to mannitol was 1:3.38. The predicted EE% of nifedipine was 94.73%, which was verified thrice, the actual EE% of nifedipine was 92.53%, RSD% = 0.71%.

### 2.3. Characterization of Liposomes

**Figure 2:**
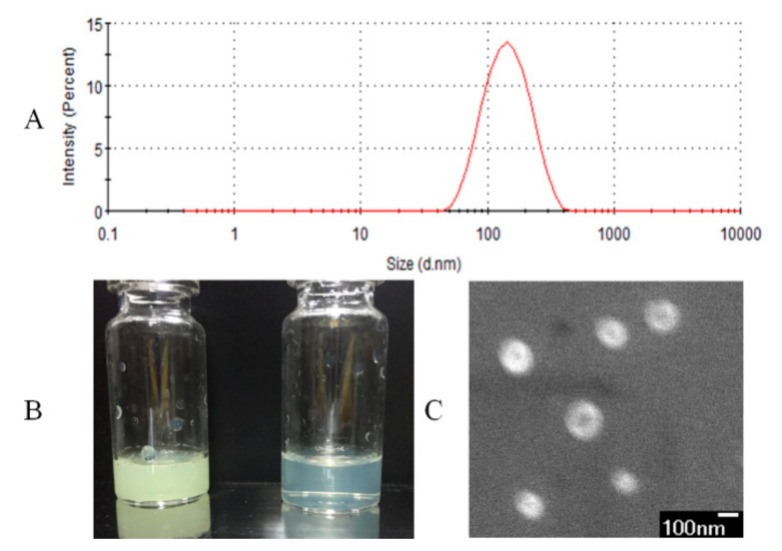
Characterization of nifedipine liposomes. (**A**) The particle size of nifedipine proliposomes after redissolving. (**B**) The appearance of nifedipine liposomes at 1.4 mg/mL (yellow) and at 140 μg/mL (canary). (**C**) SEM of nifedipine proliposomes (×33,000) at 3.0 kV.

The characteristics of nifedipine liposomes were shown in Figure 2. In Figure 2A, the particle size of reconstituted nifedipine proliposomes was 114.3 ± 7.2 nm and the zeta potential value was −4.1 ± 0.3 mV. We measured the solubility of nifedipine and nifedipine proliposomes in artificial gastric fluid, artificial intestinal fluid and water. The solubility of nifedipine was 7.1, 7.2, and 7.5 μg/mL in pH 1.2, 6.8, and 7 solutions, respectively. However, the solubility of nifedipine in proliposomes was 176.4, 91.8, and 97.9 μg/mL in artificial gastric fluid, artificial intestinal fluid, and water, respectively. The solubility of nifedipine was improved after entrapment into liposomes. In Figure 2B, the concentration of nifedipine liposomes was 1.4 mg/mL (yellow) and 140 μg/mL (canary), respectively. They all showed relatively high clarity. Spherical morphology of nifedipine liposomes was confirmed by SEM (Figure 2C). SEM image of nifedipine liposomes showed spherical with uniform size and smooth surface. If the various components in proliposomes did not form a homogenous and stable complex, the mannitol and nifedipine should show a typical crystal form. We alsoevaluated the chemical–physical characteristics of the product after spry drying. The content of nifedipine fell by 8.44% after spray-drying. The EE% of nifedipine liposomes solution before spry drying was 94.47% comparing with proliposomes of 92.53%. It means that the nifedipine proliposomes is relatively stable for spray-drying.

**Figure 3:**
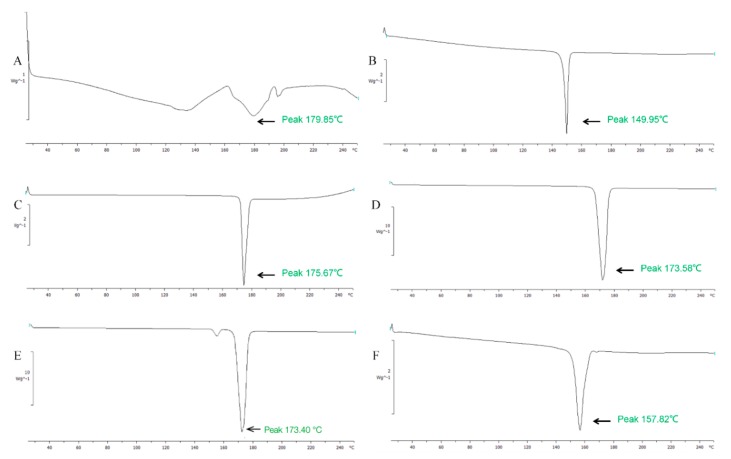
The DSC analysis of nifedipine formulations. (**A**) phospholipid, (**B**) cholesterol, (**C**) nifedipine, (**D**) mannitol, (**E**) physical mixture, and (**F**) nifedipine proliposomes.

DSC analysis was used to determine molecular states of SPC, Chol, mannitol, and nifedipine in proliposomes. The endothermic peak of SPC, Chol, mannitol, nifedipine proliposomes, and physical mixture of raw materials were shown in Figure 3. In Figure 3A, the DSC spectrum of SPC had a wide, atypical absorption peak (179.9 °C) due to it was a mixture without obvious phase transition temperature. In contrast, the DSC of Chol (Figure 3B), nifedipine (Figure 3C), and mannitol (Figure 3D) had typical single absorption peaks at 150.0 °C, 175.7 °C, and 173.6 °C, respectively. It might be caused by crystal morphology change when the temperature was at the melting point. The physical mixture samples (Figure 3E) showed endothermal peaks at 173.4 °C and 152.0 °C, the endothermal peaks were the Chol peak, mannitol peak, and nifedipine peak with no obvious change, which indicated no interaction between the various substances. But the endothermic peak of nifedipine proliposomes (Figure 3F) exhibited displacement against physical mixture. This suggested that nifedipine proliposomes changed the physical state of raw materials to form liposomes. The molecular state of proliposomes was different from simple physical mixture.

### 2.4. Drug Release Profiles

**Figure 4:**
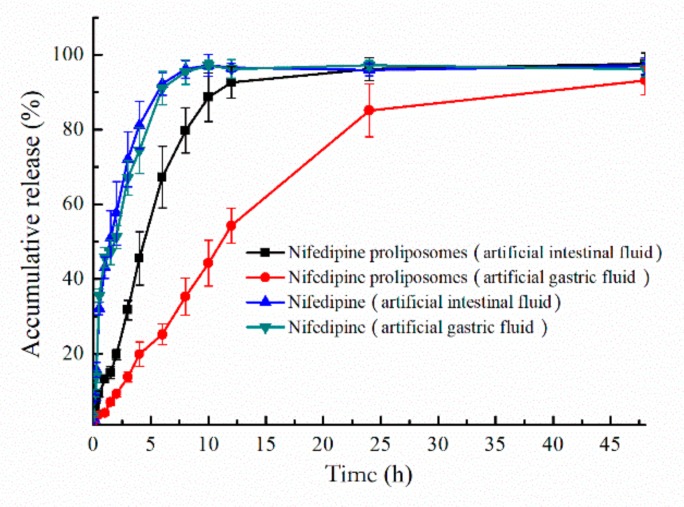
Accumulative release of nifedipine. Artificial intestinal fluid and gastric fluid were used as medium to compare the release of nifedipine and nifedipine proliposomes.

The oral preparations need to undergo different environments in the digestive tract after administration. Release behavior of nifedipine proliposomes in different parts of the digestive tract should be investigated. Two different release mediums were used to simulate the environment of the digestive tract. Release profiles of proliposomes in artificial gastric fluid (pH 1.2) and artificial intestinal fluid (pH 6.8) were shown in Figure 4. Nifedipine proliposomes had a lower release rate than nifedipine due to controlled release effect in liposomes. Free nifedipine cumulatively released approximately 81.1% and 74.3% at pH 6.8 and pH 1.2 within 4 h, respectively. On the other hand, the accumulative release of nifedipine from proliposomes was 88.7% in the artificial intestinal fluid within 10 h and 85.1% in the artificial gastric fluid within 24 h. Overall, free nifedipine released faster than proliposomes. In addition, nifedipine proliposomes had a relatively faster release rate in the artificial intestinal fluid than in artificial gastric fluid; the proliposomes seemed to be further unstable in the artificial intestinal fluid than artificial gastric fluid.

Mathematical models were used to analyze the release profiles of proliposomes in artificial gastric fluid and artificial intestinal fluid (Table 3). The regression coefficient (R^2^) was used to evaluate the degree of model fitting. First-order model could well predict the release profile of nifedipine or proliposomes releasing in artificial intestinal fluid and artificial gastric fluid (R^2^ > 0.96).

### 2.5. Nifedipine Analysis Method In Vivo

The nifedipine analysis method in plasma was assessed by calibration curve, accuracy, and precision, recovery and matrix effect. Plasma samples after administration nifedipine were analyzed by HPLC at a detection wavelength of 235 nm and nimodipine was used as an internal standard. The retention time of nifedipine and nimodipine were 7.75 min and 17.97 min, respectively. The endogenous substance in plasma did not interfere with the determination of the samples. The linear range of this method measuring nimodipine was from 25 to 3000 ng/mL; least-squares linear regression constants (R^2^) was higher than 0.99. Methodology examination of accuracy (intra-day and inter-day), precision, recovery, and matrix effect were listed in Table 4. Data of methodology showed that this method could accurately measure the plasma drug concentration of nifedipine.

### 2.6. Pharmacokinetics Study

**Figure 5:**
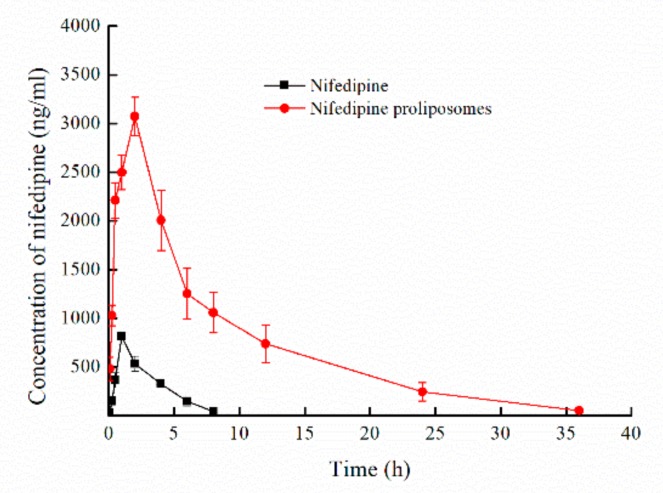
The mean plasma concentration-time profiles of nifedipine proliposomes and nifedipine (n = 5). Nifedipine proliposomes and nifedipine following oral administration in rats at a dose level of 6 mg/kg to evaluate the pharmacokinetics and bioavailability of nifedipine proliposomes.

Pharmacokinetics profiles of nifedipine and nifedipine proliposomes were evaluated after oral administration by measuring the plasma drug concentration. Mean plasma concentration-time profiles were shown in Figure 5. Main pharmacokinetic parameters of nifedipine and nifedipine proliposomes were shown in Table 5. The C_max_ of nifedipine proliposomes after oral administration was 3074 ng/mL, which was 3.76-fold greater than oral nifedipine (818 ng/mL). Nifedipine proliposomes had a durable and smooth plasma drug concentration, and the changing trend of plasma drug concentration after oral nifedipine proliposomes was slower comparing to oral nifedipine, which could reduce side effect due to variations in drug concentration. Elimination half-life (T_1/2__β_) of proliposomes had prolonged 4.21 times than oral nifedipine. Other parameters (MRT, CL) also showed that oral nifedipine was cleared faster than proliposomes. Area under the concentration-time curve (AUC_0–∞_) values of nifedipine proliposomes and oral nifedipine were 26,970.80 and 2673.97 ng × h/mL, proliposomes improved 10.09 times of AUC_0–∞_ than oral administration free nifedipine, which meant that the relative bioavailability of oral nifedipine was 9.91% comparing with nifedipine proliposomes. The above data showed that control release preparation of proliposomes could reduce plasma drug concentration fluctuations and enhance bioavailability.

Membrane permeability and dissolution behavior are two key parameters for influencing drug oral bioavailability. Bioavailability of oral drugs within the gastrointestinal tract is not only related to absorption rate, but also water solubility. Yuka Funakoshi measured the absolute bioavailability using nifedipine solution for intravenous (containing 1.0% polysorbate 80) and nifedipine suspensions for oral: ~8.5% at dose of 1 mg/kg. However, the AUC of oral nifedipine solution was 5.43 times than oral nifedipine suspensions [19]. Slow dissolution of nifedipine crystal type in enteric canal is an important factor of low bioavailability. In addition, proliposomes could extend the elimination half-life of nifedipine from 1.57 h to 6.61 h, which could reduce drug concentration depended unwanted side effects (e.g. intraocular pressure decrease, headache, and bradycardia).

Why did proliposomes improve the pharmacokinetics of nifedipine? When proliposomes were given by oral administration, proliposomes were hydrated into liposomes in the stomach and liposomes were then absorbed by the small intestine and lymphatic system through active transport and passive absorption [20]. The prerequisite of drug absorption in the intestine was that drug should be in dissolved state, only dissolved drugs could penetrate intestinal epithelial cells. Absorption of lipophilic drugs in intestinal was limited by their restricted dissolution rate. Absorption increasing of proliposomes could be attributed to enhance contact level of drug and the intestinal membrane [14]. Proliposomes could improve the solubility of nifedipine and increase the contact area between the drug and intestinal epithelial cells to improve absorption efficiency.

On the other hand, liposomes were principally absorbed within the intestinal. Liposomes formed colloidal structures to delay in gastric emptying for improving residence time. This phenomenon was beneficial for enhancing bioavailability and stabilizing plasma drug concentration.

Improved bioavailability of nifedipine proliposomes might be caused by intestinal lymphatic transport pathway. It was a one-way channel from the lymphatic system to the blood [14]. Elgart et al. found that nanoparticles were taken up in the proximal small intestine by phagocytosis of chylomicron pathway to enter the lymphatic system, which could avoid first-pass metabolism by liver to improve bioavailability [20]. Nifedipine proliposomes exhibited a longer elimination half-life, which phenomenon might be due to the transfer speed of lymph was lower than blood. In conclusion, proliposomes made a great contribution to improving the bioavailability of nifedipine after oral administration through control release, improving solubility, enhancing adhesion in gastrointestinal mucosa, stimulating lymphatic absorption, and avoiding liver metabolism.

## 3. Materials and Methods

### 3.1. Materials and Animals

Soybean lecithin (SPC) was purchased from Shanghai Jinban Pharmaceutic Co., Ltd. (Shanghai, China). Cholesterol (Chol) was obtained from Beijing Dingguo Changsheng Biotechnology Co., Ltd. (Beijing, China). Nifedipine and nimodipine were purchased from Shanghai Yuanye Bio-Technology Co., Ltd. (Shanghai, China). Other commonly used organic reagents are analytically pure. Wistar rats were purchased from Liaoning Changsheng Biotechnology Co., Ltd. (Shenyang, China).

### 3.2. Preparation Proliposomes

Methanol injection combined with spray-drying was used to prepare nifedipine proliposomes. Briefly, SPC, Chol, and nifedipine were dissolved in methanol. This solution was then injected into water at a volume ratio of 1:8 to prepare liposomes. Then, the liposomes were subjected to spray drying to obtain proliposomes at an inlet temperature of 130 °C. Mannitol was selected as a protective carrier. The nifedipine proliposomes was stored at 4 °C in darkness.

### 3.3. Optimization of Proliposomes Formulation

Entrapment efficiency is an important parameter to evaluate Nano-drug. Based on single-factor experiments, three related factors—*X1* (the mass ratio of drug-to-SPC), *X2* (the mass ratio of SPC-to-Chol), and *X3* (the amount of SPC)—were used to optimize the formulation of proliposomes with EE% as the evaluation index. Factor-coded levels were listed in Table 6. Fifteen groups of experiments were identified through Box–Behnken design (BBD) with three-factor and three-coded level. The interaction between two factors was described through response surface plot. Finally, curve-fitting was used to generate equation to calculate the optimal formulation of nifedipine proliposomes.

The EE% of nifedipine liposomes was determined by ultracentrifugation method. Briefly, nifedipine proliposomes was reconstituted into liposome suspension with water. The total nifedipine in proliposomes (W_t_) was measured by high-performance liquid chromatography (HPLC) after methanol demulsification to release nifedipine. Then, the nifedipine liposomes, after resuspending, were used to separate free nifedipine (W_f_) on an Optima MAX-XP ultracentrifuge (Beckman Coulter, Inc. Brea, CA, USA). Finally, nifedipine concentration was determined on an Agilent SB-C18 HPLC column (4.6 mm × 250 mm, 5 µm) running with acetonitrile/water (50:50, *v*/*v*) of 1 mL/min at a detection wavelength of 235 nm [21].

EE (%) was calculated using the equation:

EE% = (1 − W_f_/W_t_) × 100%

The solubility of nifedipine and nifedipine proliposomes in different solutions were measured, including artificial gastric fluid, artificial intestinal fluid, and water at 37 °C. The content changing of nifedipine after spray drying was evaluated.

### 3.4. Characterization of Proliposomes

Nifedipine proliposomes was reconstituted to form liposomes by water. Size and zeta potential of liposomes were measured by Zetasizer Nano ZS 90 from Malvern Instruments, Ltd. (Malvern, UK). The size distribution was surveyed for 120 s in intensity-weighted Gaussian distribution mode with the dynamic light scattering method.

Morphology of nifedipine proliposomes was analyzed on a JEOL field emission JSM-6700F scanning electron microscope (SEM) (Tokyo, Japan) at 3 kV accelerating voltage. Proliposomes used in SEM study were redissolved and directly applied onto a clean silicon slice. The sample was allowed to dry at room temperature overnight. SEM image was obtained in the secondary electron mode.

A Mettler differential scanning calorimetry (DSC) STARe system (Mettler-Toledo, Columbus, OH, USA) was used to analyze the proliposomes. Nifedipine, SPC, mannitol, Chol, nifedipine proliposomes, and physical mixture of raw material were sealed into aluminum pans and analyzed. Samples were heated from 25 °C to 250 °C the a 10 °C/min rate of temperature rise to obtain thermograms.

### 3.5. Drug Release Profiles

We used two different release mediums to simulate the release behavior of nifedipine from proliposomes under different conditions. In brief, nifedipine proliposomes reconstituted suspension was sealed into dialysis bags (molecular weight cut-off: 8000–12,000 Dalton), which were placed in the release medium containing 0.5% Tween 80 to increase the solubility of nifedipine. We used two different mediums to simulate gastric fluid (pH = 1.5, containing 10 μg/mL of pepsin) and simulated intestinal fluid (pH = 6.8, containing 10 μg/mL of trypsin and 0.05 M of KH_2_PO_4_) to evaluate the cumulative release rate of nifedipine [22]. At predetermined time points, 1 mL of external dialysis medium was collected and replaced by an equal volume of fresh medium. The samples were analyzed by HPLC method. Four basic release models (zero-order: Mt/M∞ = kt, first-order: ln (1 − Mt/M∞) = −kt, Higuchi: Mt/M∞ = kt^1/2^, Korsmeyer − Peppas: Mt/M∞ = kt^n^) were used to analyze the obtained data. Mt was the release amount of nifedipine at the time point of t. M∞ was the maximum value of release nifedipine.

### 3.6. Pharmacokinetic Study

Animal experiments were approved by Institutional Animal Care and Use Committee of Jilin University (No. 201703052). A total of 10 Wistar rats weighing 275 ± 10 g were randomly assigned to two groups. The rats were maintained on a normal diet and were placed on fasting with free access to drinking water before study. One group was given oral a single dose nifedipine of 6 mg/kg, the other group received oral proliposomes containing 6 mg/kg nifedipine. Blood samples were drawn into heparin sodium treated EP tube from retro-orbital sinus at 5, 15, 30 min, 1, 2, 4, 6, 8, 12, 24, and 36 h. The blood was immediately centrifugated at 10,000 rpm for 5 min to obtain plasma. The pharmacokinetic parameters were calculated using WinNonlin version 5.2 (Pharsight Co., Mountain View, CA, USA) by non-compartment model. The pharmacokinetic parameters obtained by fitting non-compartment model and plasma drug concentration of nifedipine.

The concentration of nifedipine in plasma was measured by HPLC method. Briefly, 100 μL plasma samples and 10 μL of 20 μg/mL internal standard nimodipine were stirred for 30 s, 10 μL of 1 mol/L NaOH was then added into the sample with stirring for 1 min [23]. The analytes were extracted from the samples with 1 mL of diethyl ether/chloroform (5:1 v/v) by vortex mixing for 5 min. The organic phase was separated by centrifugation at 10,000 rpm for 10 min. The extract was dried by nitrogen at 37 °C, and the residue was reconstituted with 200 μL mobile phase and injected into HPLC system to analyze.

### 3.7. Statistical Analysis

The data were analyzed for statistical significance using Student’s *t*-test and *p*-values < 0.05 were regarded as significant. All data were expressed as mean ± SD.

## 4. Conclusions

In this study, we designed nifedipine proliposomes through a spray-drying method and optimized the formulation to obtain a good EE%. We found that nifedipine proliposomes could significantly improve the bioavailability based on the pharmacokinetic study. But the mechanism of enhanced absorption was not clear.

In future work, we plan to study the pharmacokinetic–pharmacodynamic relationship of nifedipine proliposomes and clearly delineate the mechanism of enhanced oral absorption.

## Figures and Tables

**Table 1:** The experimental conditions and the corresponding results.

Run	Levels of Independent Factors			Response EE%
	*X1*	*X2*	*X3*	
1	−1	−1	0	82.06
2	1	−1	0	72.95
3	−1	1	0	81.37
4	1	1	0	73.27
5	−1	0	−1	85.79
6	1	0	−1	89.12
7	−1	0	1	91.02
8	1	0	1	71.93
9	0	−1	−1	78.85
10	0	1	−1	81.36
11	0	−1	1	78.15
12	0	1	1	73.15
13	0	0	0	94.12
14	0	0	0	93.41
15	0	0	0	94.05

**Table 2:** Statistical analysis for mathematical model and variance.

Source	*df*	Sum of Squares	Mean Square	F Value	*p*-Value Prob > F	
Model	9	922.61	102.51	232.19	<0.0001	Significant
*X1*	1	136.04	136.04	308.14	<0.0001	
*X2*	1	0.99	0.99	2.24	0.1951	
*X3*	1	54.6	54.6	123.67	0.0001	
*X1X2*	1	0.26	0.26	0.58	0.4815	
*X1X3*	1	125.44	125.44	284.13	<0.0001	
*X2X3*	1	13.91	13.91	31.51	0.0025	
*X1* ^2^	1	89.6	89.6	202.96	<0.0001	
*X2* ^2^	1	490.11	490.11	1110.12	<0.0001	
*X3* ^2^	1	73.90	73.90	167.38	<0.0001	
Residual	5	2.21	0.44			
Lack of fit	3	1.90	0.63	4.14	0.2007	No Significant
Pure error	2	0.31	0.15			
Coe. total	14	924.81				

R^2^ = 0.9976, R_Adj_^2^ = 0.9933, R_Pred_^2^ = 0.9664.

**Table 3:** The regression coefficient of different release model of nifedipine in artificial gastric fluid and artificial intestinal fluid.

	Time	Medium	Regression Coefficient (R^2^)			
			Zero-Order	First-Order	Higuchi	Korsmeyer–Peppas
Nifedipine	0–48 h	artificial intestinal fluid	−1.165	0.985	0.340	0.790
	0–48 h	artificial gastric fluid	−1.116	0.966	0.378	0.806
Proliposomes	0–48 h	artificial intestinal fluid	0.149	0.980	0.799	0.827
	0–48 h	artificial gastric fluid	0.774	0.988	0.924	0.943

**Table 4:** Validation of the nimodipine determination method in plasma.

	LQC (75 ng/mL)	MQC (500 ng/mL)	HQC (2000 ng/mL)	IS (2000 ng/mL)
Intra-day precision (RSD%) (n = 6)	8.55	5.81	10.87	
Inter-day precision (RSD%) (n = 6)	8.70	5.81	7.12	
Accuracy (RE%) (n = 6)	9.26	9.44	5.41	
Recovery/RSD% (n = 6)	83.37/2.67	83.90/2.86	83.11/2.73	88.59/4.11
Matrix effect/RSD% (n = 5)	87.40/4.50	87.85/3.99	88.76/2.38	91.05/2.98

**Table 5:** Pharmacokinetic parameters of nifedipine in rats following an oral of nifedipine or nifedipine proliposomes, respectively (mean ± SD, n = 5).

Parameters	Nifedipine	Nifedipine Proliposomes
C_max_ (ng/mL)	818.20 ± 83.19	3074.20 ± 196.77 **
T_max_ (h)	1.00	2.00
T_1/2β_	1.57 ± 0.18	6.61 ± 0.49 **
AUC_0–∞_ (ng × h/mL)	2673.97 ± 175.06	26970.80 ± 4650.71 **
MRT	3.0 ± 0.12	8.95 ± 0.90 **
V (mL)	1522.41 ± 223.56	646.32 ± 93.63 **
CL (mL/h)	671.89 ± 38.24	68.12 ± 10.96 **

** *p* < 0.01.

**Table 6:** Levels of factors used in Box–Behnken design (BBD).

Factors	Range and Level		
	−1	0	1
*X1* (the mass ratio of drug-to-SPC)	0.5:1	1:1	1.5:1
*X2* (the mass ratio of SPC-to-Chol)	2:1	4:1	6:1
*X3* (the concentration of SPC)	12 mg/mL	14 mg/mL	16 mg/mL
